# Genetic markers of children asthma: predisposition to disease course variants

**DOI:** 10.18699/VJGB-23-47

**Published:** 2023-07

**Authors:** M.V. Smolnikova, Ed.W. Kasparov, M.A. Malinchik, K.V. Kopylova

**Affiliations:** Scientific Research Institute of Medical Problems of the North – a separate division of the Federal Research Center “Krasnoyarsk Science Center” of the Siberian Branch of the Russian Academy of Sciences, Krasnoyarsk, Russia; Scientific Research Institute of Medical Problems of the North – a separate division of the Federal Research Center “Krasnoyarsk Science Center” of the Siberian Branch of the Russian Academy of Sciences, Krasnoyarsk, Russia; Scientific Research Institute of Medical Problems of the North – a separate division of the Federal Research Center “Krasnoyarsk Science Center” of the Siberian Branch of the Russian Academy of Sciences, Krasnoyarsk, Russia; Scientific Research Institute of Medical Problems of the North – a separate division of the Federal Research Center “Krasnoyarsk Science Center” of the Siberian Branch of the Russian Academy of Sciences, Krasnoyarsk, Russia

**Keywords:** asthma, cytokine, gene polymorphism, child, asthma severity, level of diseases control, бронхиальная астма, цитокин, полиморфизм генов, дети, степень тяжести астмы, уровень контроля

## Abstract

Asthma is a heterogeneous and often difficult to treat condition that results in a disproportionate cost to healthcare systems. Children with severe asthma are at increased risk for adverse outcomes including medication-related side effects, life-threatening exacerbations, and impaired quality of life. An important therapeutic focus is to achieve disease control, which is supposed to involve a personalized approach to treatment of asthma of any severity. Asthma is a multifactorial disease with a significant genetic determinant, however, the inheritance of asthma has not been fully elucidated. Polymorphic genes of inflammatory mediators, including cytokines, play an important role in developing various disease forms. In the current study, large-scale original data on the prevalence of cytokine gene genotypes (IL2, IL4, IL5, IL6, IL10, IL12, IL13, IL17A, IL31, IL33, IFNG, TNFA) among Russian children with asthma in Krasnoyarsk region have been obtained. Genotyping was carried out using real-time PCR. We identified markers predisposing to the development of different variants of the course of childhood asthma: the CT genotype and T allele of IL4 rs2243250 are associated with asthma (p < 0.05), especially in mild asthma and in controlled asthma. The TT genotype and allele T of IL13 rs1800925 are associated with severe and uncontrolled asthma (p < 0.05). The AA genotype of IL17A rs2275913, the TT genotype of IFNG rs2069705 and allelic A variants of TNFA rs1800629 are associated with mild asthma, and the TT genotype of IFNG rs2069705 is additionally associated with controlled asthma. The results obtained will supplement information on the prevalence of polymorphic variants of the cytokine genes in the Russian population and in asthma patients with different disease courses, which is likely to be used in order to shape a plan for Public Health Authority to prevent the development of severe uncontrolled asthma and to optimize personalized therapy.

## Abstract (ru)

Астма – хроническое гетерогенное и часто трудно поддающееся лечению состояние, приводящее к несоразмерным расходам системы здравоохранения. Дети с тяжелой астмой подвержены повышенному риску неблагоприятных исходов, включая побочные эффекты, связанные с приемом лекарств, угрожающие жизни обострения и ухудшение качества жизни. Важным терапевтическим акцентом является достижение контроля над заболеванием, что подразумевает персонифицированный подход к лечению при любой степени тяжести астмы. Астма относится к мультифакториальным заболеваниям, имеющим значимую генетическую детерминанту, однако наследование астмы на сегодняшний день полностью не объяснено. В развитии разных форм заболевания особую роль играют полиморфные гены медиаторов воспаления, в том числе цитокинов. В настоящем исследовании впервые получены масштабные данные о распределении генотипов генов цитокинов (IL2, IL4, IL5, IL6, IL10, IL12, IL13, IL17A, IL31, IL33, IFNG, TNFA) среди больных астмой русских детей Красноярского края. Генотипирование осуществлено с использованием метода полимеразной цепной реакции в режиме реального времени (ПЦР-РВ). В ходе исследования нами выявлены маркеры, предрасполагающие к развитию различных вариантов течения астмы у детей: генотип CT и аллель Т rs2243250 IL4 ассоциированы с развитием астмы ( p < 0.05), особенно при легкой форме и контролируемом течении. Генотип ТT и аллель Т rs1800925 IL13 ассоциированы с астмой, в том числе тяжелой степени, и с неконтролируемой формой ( p < 0.05). Установлено, что генотипы АА IL17A rs2275913, ТТ IFNG rs2069705 и аллельный вариант А TNFA rs1800629 ассоциированы с легкой степенью астмы, генотип ТТ IFNG rs2069705 ассоциирован также с контролируемой формой. Получен- ные результаты дополнят данные о характеристике распределения полиморфных вариантов генов цитокинов в русской популяции и у больных астмой с различным течением заболевания, что можно будет использовать для формирования планов органов практического здравоохранения в отношении профилактики развития тяжелой неконтролируемой астмы и в целях оптимизации персонифицированной терапии

## Introduction

Asthma is one of the most common diseases of the lower respiratory tract; it is a heterogeneous disease characterized by airway inflammation and hyperactivity. Asthma most often begins in early childhood, has a variable course and an unstable phenotype progressing over time (Hancox et al., 2012). It significantly limits and worsens the quality of human life in case of uncontrolled and severe disease. According to WHO estimates, asthma annually leads to the loss of 26.2 million in the world as measured by DALYs (disability-adjusted life years – an indicator of healthy life lost due to disability), which is 1 % of the total global burden of disease (GBD 2015 Chronic Respiratory Disease Collaborators…, 2017). Today, asthma is a global health problem of great socio-economic importance, i. e. about 339 million people worldwide suffer from asthma. The increase in the prevalence and incidence of asthma worldwide is influenced by both genetic background and a large number of environmental factors included in the “modern lifestyle” concept. Moreover, asthma prevalence, severity, and mortality vary greatly by ethno-geographic origin.

Based on expert estimates, the number of asthma patients in Russia exceeds official figures, i. e. according to their calculations, 5.9 million instead of 1.3 million people suffer from asthma in our country. In addition, according to the reported data, since asthma is a disabling and dangerous disease, about 41 % of asthma patients receive a disability pension. The prevalence of the disease among adults is 6–7 %, among children and adolescents it is 8–10 %, exceeding the incidence rate of cardiovascular disease, breast cancer and HIV infection (Chuchalin et al., 2014). According to 2020 data, more than 42.5 thousand asthma individuals were recorded in Krasnoyarsk region, including both adults and children. The prevalence among adolescents has been noted to be steadily increasing

There are a number of asthma phenotypes and endotypes. Asthma classification is to group patients based on observable combinations of clinical, biological and physiological characteristics into so-called phenotypes. Simply stated, phenotypes are defined as “observable characteristics resulting from a combination of hereditary and environmental influences” (Wenzel, 2012). It is important to emphasize that the phenotype of asthma can change over time, which is caused by environmental factors, allergens, seasonal changes, respiratory infections, iGCS (inhalant glucocorticosteroids) therapy, etc. Asthma is known to be classified according to the Global Initiative for Asthma (GINA) and both severity and level of control as well.

Asthma severity is associated with the intensity of the pathological process and it is possible to correctly determine its degree before treatment, since there is a decrease in symptoms with effective therapy. According to the recommendations of the GINA working group, the asthma severity can be distinguished as: intermittent, persistent-mild, persistent-moderate, and persistent-severe. Level of disease control is the degree to which symptoms and functional limitations are controlled, as well as the minimization of risks of asthma exacerbation, and the prevention of deterioration in lung function with medical treatment. Whatever the disease severity, the goal for patients is to have well-controlled asthma. According to the degree of control, it is classified into controlled and uncontrolled asthma

An important characteristic of asthma is the multifactorial nature of the disease, with the pathogenesis of development combining both genetic and environmental factors. Extrinsic factors are sure to be numerous and responsible for the activation of asthma manifestation or cause its exacerbation. The internal characteristics of the individual are of greatest importance. Intrinsic (congenital) factors include genetic predisposition, gender and ethnic origin. The important role of heredity in asthma occurrence has been confirmed by family, twin, and genetic epidemiological studies (Thomsen, 2014).

The genetic component of the disease is provided by the combined action of various groups of genes. The same asthma phenotype in different individuals may result from the “breakdown” of various genes; the disease development might result from a mutation of several genes at once in every single individual. In addition, not only the possibility of developing the disease, but also its severity, response to therapy, etc. are determined by hereditary factors

Airway inflammation underlying asthma is also caused by the so-called cytokine network, which is a self-regulating system; when its functionality is impaired, an excess or insufficient production of various cytokines takes place, which turns out to result in the development of pathological processes. More than 50 cytokines are known to be involved in the immune pathogenesis of this disease, and the role of each of those has not been fully elucidated. Significantly higher levels of cytokines such as GM-CSF, IL-4, IL-5, IL-10, IL-12, IL-13, IL-17A, IL-8, IL-18, TNF-α can be detected in serum.

Gene polymorphism is known to cause differences in the expression and level of protein production. Currently, a huge number of polymorphic regions have been identified in the genes of a number of cytokines and their receptors. Despite the progress made in the study of the immune-pathogenesis of asthma, there has been no agreement of opinion on the pathogenetic role of polymorphic variants for cytokine genes related to asthma development as well as its phenotypes, which is to be further studied. In addition, there have been some contradictions in the study results for different populations worldwide, as the frequency distribution of polymorphic variants of genes, including cytokine ones, has unique features depending on ethno-geographic characteristics (Puzyrev et al., 2007). Therefore, a comparative analysis of genetic parameters in a single population in order to identify risk factors for asthma phenotype development is to be relevant.

Thus, asthma is the subject of research aimed at studying the disease process, the role of various mediators (including cytokines), treatment approaches, and the role of the genetic determinant as well.

The aim of the study was to identify markers for the development of various asthma phenotypes in Russian children of Krasnoyarsk region.

## Materials and methods

Asthma patients (n = 317) and healthy children (control group) (n = 229) matched by sex, age and ethnicity were the object of the study. Criteria for patients to be involved in the study were the following: an established diagnosis of bronchial asthma; age from 8 to 18 years; more than one-year of asthma experience; both parents of the child being Russians. There are some criteria for exclusion from the study such as concomitant decompensated diseases as well as for inclusion in the control group: the absence of allergic pathologies and bronchopulmonary diseases; age from 6 to 18 years; both parents of the child being Russians.

Depending on the severity of the disease, determined in accordance with the recommendations of the GINA working group (Global Initiative for Asthma, updated, 2018 and 2021), the following groups were distinguished: intermittent, persistent-mild, persistent-moderate, and persistent-severe. In the course of the study, we grouped patients according to the following severity levels: intermittent asthma and persistent-mild asthma into the “mild” asthma group (n = 131), persistent-moderate and persistent-severe into the “severe” asthma group (n = 186) due to the small number of patients in some groups. Depending on the level of disease control, based on the results of the asthma control test in children (C-ACT, asthma control test), the following groups were distinguished: controlled asthma (n = 171) – 20 or more points, and uncontrolled asthma – less than 19 points (n = 146).

The work was performed in accordance with the principles stated in the Declaration of Helsinki on research in humans and animals. The studies were approved at a meeting of the local ethical committee of Scientific Research Institute of Medical Problems of the North (The Minutes No. 12 dated December 10, 2013). The examination protocol for patients and healthy children (control group) met ethical standards and was approved by the Biomedical Ethics Committee of Scientific Research Institute of Medical Problems of the North. The right to conduct an examination was legally secured by the informed written consent of the parent

DNA extraction from blood was carried out using the DIAtom™ DNA Prep100 reagent kit (Isogene, Russia). Genotyping was conducted by the real-time PCR method using specific oligonucleotide primers and fluorescently labeled probes according to the manufacturer’s protocol (DNA Synthesis, Russia) and the Rotor-Gene Q 6 plex instrument (QIAGEN, Germany).

Comparison of allele and genotype frequencies between groups was performed using an online calculator https://med statistic.ru/. The χ2 test was used to assess the association of a trait-genotype with the disease in groups of sick and almost healthy children. The threshold significance level was taken equal to 0.05. The odds ratio (OR) was used with a 95 % confidence interval (CI) for an assessment of the degree of association of genetic markers with traits.

## Results

In order to identify genetic markers of asthma, a comparative analysis of the frequency of single nucleotide polymorphisms (SNPs) between patients and children in the control group was made. Comparative analysis of allele and genotype frequencies between the cohort of asthma patients and controls revealed statistically significant differences in the SNPs distribution in the promoter regions of IL4 rs2243250 and IL13 rs1800925 (Table 1).

**Table 1.**
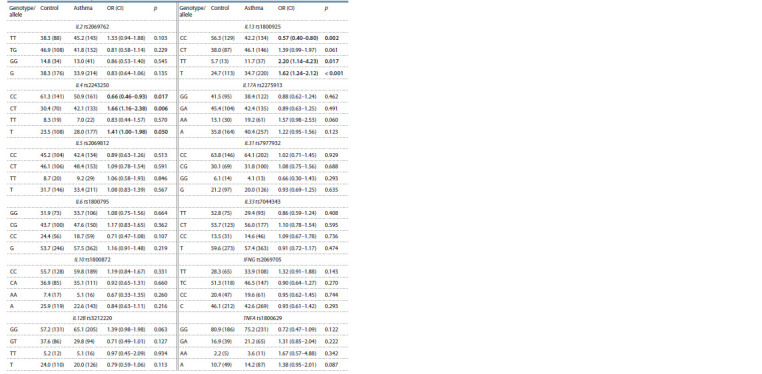
Prevalence of the genotypes and alleles of the SNPs in asthma patients and control group, % (n)

The prevalence of the IL4 rs2243250 T allele in the group of asthma patients relative to the control group was shown (28 % versus 23.5 %, p = 0.05), with the frequency of the heterozygous CT genotype of IL4 rs2243250 being also statistically significantly higher in asthma patients compared to the control group (p = 0.006). The frequencies of the TT genotype and T allele of IL13 rs1800925 are significantly higher in the group of patients relative to the control group ( p <0.05).

A comparison of the genotype and allele frequencies in the group of asthma patients depending on the severity and level of asthma control was made (Tables 2 and 3) to study in detail the association of allelic variants of cytokine genes with the characteristics of asthma development

**Table 2.**
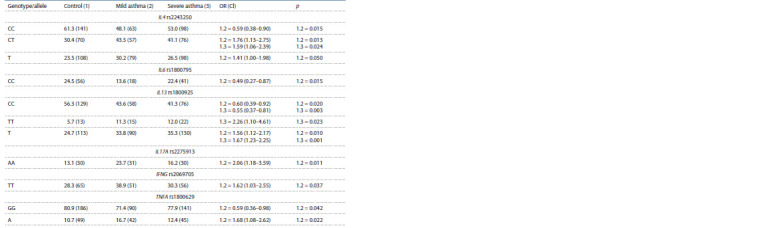
Prevalence of the genotypes and alleles of the SNPs in patients with mild and severe asthma and in control group, % (n) Notе. Genotypes and alleles are shown, with their frequency difference between the comparison groups р ≤ 0.05.

**Table 3.**
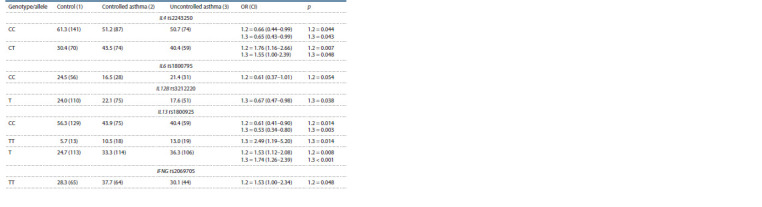
Prevalence of the genotypes and alleles of the SNPs in patients with controlled and uncontrolled asthma and in control group, % (n) Notе. SNPs are given, with the frequency difference between the comparison groups р ≤ 0.05.

As a result of analysis of the IL4 rs2243250 and IL13 rs1800925 distribution depending on the severity of asthma, a high frequency of both the CT genotypes of IL4 rs2243250 and TT genotypes of IL13 rs1800925 in the group with severe asthma relative to the control group was noted, and for the СТ genotype of IL4 rs2243250, in the group of children with mild asthma ( p <0.05). Analysis of the allele frequencies of these polymorphic gene variants revealed significant differences in the rare T allele frequency of IL4 rs2243250 and IL13 rs1800925 in children with mild and severe asthma (in the case of rs1800925) compared with healthy children ( p < 0.05).

When comparing the frequency of IL17A rs2275913, IFNG rs2069705 and TNFA rs1800629 genotypes and alleles, AA homozygotes of rs2275913 ( p = 0.01), TT of rs2069705 ( p = 0.03) and allelic A variant of TNFA rs1800629 were shown to be significantly more common in the group of children with mild asthma relative to the control group.

As a result of the analysis of the IL4 rs2243250 and IL13 rs1800925 distribution depending on the level of asthma control, it was demonstrated that the CT of IL4 rs2243250 and TT of IL13 rs1800925 genotypes are more common in the uncontrolled asthma group compared to the controls ( p < 0.05). The CT genotype of IL4 rs2243250 is also significantly more common in controlled asthma patients than in controls. Allele frequency analysis revealed the differences in the frequency of the rare T allele of IL13 rs1800925 between groups of children with both controlled and uncontrolled asthma, and healthy children as well ( p < 0.05). When comparing the frequency of the IFNG rs2069705 genotypes, the homozygous TT was shown to be significantly higher in the controlled asthma group compared to the control group ( p < 0.05).

## Discussion

Since the inflammatory response regulation for asthma has been carried out using mediators/cytokines, the mechanisms of violation of their functionality have to be studied. The level of cytokine concentration in blood serum is affected by genetic polymorphism of the cytokine network, which turns out to have an effect on the asthma progression type. By 2022, about 1500 genes have been studied for asthma, including cytokines and their receptors (according to Phenopedia). The influence of various genes on the formation of a genetic predisposition to asthma should be noted to be significantly different in various populations, i. e. has some ethnogeographic features. Hence, there are some conflicting data in the studies on the role of genetic factors in the asthma pathogenesis. As a result of the 1000 Genomes project (http://www.1000genomes.org), data on a number of SNPs in genes, including those in promoter, exons, and intron regions have been obtained. However, there are few functional polymorphic variants (affecting the protein functions or structure) with their contribution to the pathology of asthma being ambiguous.

In our study the SNP allele and genotype frequencies of key cytokines produced by different types of the immune system cells that mediate inflammatory reactions in diseases among Russian children in Krasnoyarsk region were studied. We found significant differences in the frequency of polymorphic distribution of cytokine genes between asthma patients and the control group, allowing us to identify genetic markers that are suggestive risk factors for developing asthma, i. e. the heterozygous CT genotype and the T allele of IL4 rs2243250, the homozygous TT variant and the T allele of IL13 rs1800925.

As mentioned above, the disease course prediction, effectiveness of treatment, controlled course, prevention of the severe asthma development, as well as providing personalized therapy and asthma prophylaxis are of the greatest importance.

In order to find genetic markers of different types of asthma, we analyzed the SNPs distribution of cytokine genes in patients with different severity and control of the disease. The CT genotype and the T allele of IL4 rs2243250 was found to be associated with mild asthma, the CT genotype of IL4 rs2243250 was also associated with severe asthma; moreover, the TT genotype of IL13 rs1800925 was associated with severe asthma, and the T allele of IL13 rs1800925 – with both mild and severe asthma. Genetic markers predisposing to different forms of asthma depending on the control were also identified, i. e. both the CT genotype of IL4 rs2243250 and the T allele of IL13 rs1800925 were associated with both controlled and uncontrolled asthma, with the TT genotype of IL13 rs1800925 being associated with with uncontrolled one.

The IL4 and IL13 genes are located in one cluster of chromosome 5q31.1 and encode cytokines that play a key role in the asthma pathogenesis, namely, IL-4 and IL-13 promote airway eosinophilia, mucus hyperproduction, bronchial hyperreactivity, and IgE synthesis (Zhang et al., 2015). The SNP (rs2243250) in the IL4 promoter is associated with increased expression and production of IL-4, and SNP IL13 rs1800925 enhances the expression of IL-13 in Th2 cells. Аsthma patients with an elevated IgE level were reported to have a homozygous genotype for the rare allele T of IL4 rs2243250. Our data obtained as a result of analysis of the frequency distribution of genotypes and alleles rs2243250 and rs1800925 in Krasnoyarsk children are consistent with the study results of other scientists.

It was previously determined that the CT genotype of IL4 rs2243250 predominates in the group of Russian children with atopic asthma, and Arab asthma patients having this genotype were also found to have the highest incidence of eczema compared to the patients with the TT genotype (Hijazi, Haider, 2000; Smirnova et al., 2018). In the asthma children group, an increased incidence rate of the TC and TT genotypes of the IL4 (C-590T) polymorphism (rs2243250) compared to healthy ones was shown (Prosekova et al., 2020). W. Nie et al., in the meta-analysis including 40 studies, concluded that the CT vs. CC was significantly associated with an increased risk of developing asthma. In addition, when analyzed by ethnicity, significant associations were found in Asians and Caucasians, but not in African Americans (Nie et al., 2013). However, some studies obtained different results, for instance, the analysis of genotypes associated with asthma for C-589T of the IL4 gene did not reveal statistically significant differences between the control group and the group of asthma patients, which might be due to the small number of studied samples (Rudenko et al., 2021). And in a meta-analysis carried out by Chinese scientists, a rare allele was said to be a weak risk factor for asthma development in Caucasians (Liu et al., 2012).

Z. Liu et al. (2014) have shown that the CT and TT genotypes of IL13 rs1800925 were more common in the group of asthma patients. Scientists from Malaysia have found out that the percentage of the minor T allele in asthma patients was above the frequency of the same allele in the control, being a risk factor for the development of this pathology (Radhakrishnan et al., 2013). However, the study results on a population of children in Costa Rica have demonstrated that the T allele rs1800925 led to the progression of asthma only in children taking corticosteroids and was not associated with the risk of developing the disease (Hunninghake et al., 2007).

As a result of meta-analysis, the mutation rs1800925 was associated with an increased risk of developing asthma only in the Caucasian population, and not associated with a predisposition to asthma in Asians (Omraninava et al., 2020).

There are also controversial data, which are likely to be related to the small number of studied samples, in particular, an analysis of the distribution of alleles and genotypes of IL13 rs1800925 did not reveal statistically significant differences between the control and the group of asthma patients. However, there was a tendency to increase the proportion of allele C in the group of asthma patients (Kutlina et al., 2018). Nevertheless, polymorphic variants of the TNFA, IL4, and IL13 cytokine genes have been shown to contribute to the formation of a genetic predisposition to asthma in the Republic of Bashkortostan (Karunas et al., 2012).

While working, we have also found that the AA genotype of IL17A rs2275913, the TT genotype of IFNG rs2069705, and the A allele of TNFA rs1800629 were associated with mild asthma, and the TT genotype of IFNG rs2069705 – with controlled asthma.

The literature available on the association of IL17A rs2275913, localized in the promoter region, with the expression level and cytokine activity of IL-17A are very inconsistent. Thus, an association between the SNP and susceptibility to asthma in children has been noted, i. e. the GG genotype patients have mild to moderate asthma and low levels of IL-17A (Maalmi et al., 2014). The A allele of rs2275913 has been reported to increase the activity of the IL17A promoter and upregulate its transcription, leading to increased airway inflammation (Espinoza et al., 2011). However, another study failed to find an association between IL17A rs2275913 and asthma risk (Wang et al., 2011), while J. Chen et al. have demonstrated that the level of IL-17A expression in peripheral blood mononuclear cells was not affected by rs2275913 (Chen et al., 2010). One of the ethnicity-specific analysis showed that the G allele of IL17A rs2275913 was a protective factor of the asthma in Asians, with no association being found in Africans (Zhai et al., 2018).

It is known that one of the key Th1-cytokines is IFN-γ, involved in the many features regulation of asthma pathogenesis, including suppression of the of Th2 profile cytokine release, inhibition of the recruitment of effector cells to the site of inflammation, apoptosis induction of T-cells, eosinophils, etc. Nevertheless, there have currently been a limited number of studies investigating the role of polymorphic sites in the IFNG in the pathogenesis of asthma. The G-238A mutation in the TNFA gene has been shown to reduce the risk for developing asthma, whereas the SNP G-308A (rs1800629) was associated with the development of asthma and an increase in TNF-α production (Zedan et al., 2008). The A allele of rs1800629 has also been shown to be associated with increased TNFA transcription compared to the G allele, with its frequency varying significantly between ethnic groups and being rare in Japanese (less than 3 %) (Wilson et al., 1997).

Tomsk scientists, who have been studying pathogenetics of asthma for many years, found an association of the polymorphic variant of the TNFA gene (rs1800629) with the development of asthma, namely, the AA genotype was more often indicated in the group of patients compared to the control (Zhalsanova et al., 2020). According to a series of study results, an analysis depending on ethnodemographic data is necessary. Only in this case, the obtained markers of the diseases can be used as prognostic ones.

Some researchers have distinguished not only genetic markers of the risk of developing a disease or its forms, but also some protective markers. The CC genotypes of IL4 rs2243250, IL6 rs1800795, IL13 rs1800925, as well as the GG genotype of TNFA rs1800629 were shown in our study to be protective against the development of mild asthma. It was also determined that the CC genotypes of IL4 rs2243250, IL6 rs1800795, IL13 rs1800925 and the allelic variant T of IL12B rs3212220 can be considered to be potentially protective for the development of uncontrolled asthma.

## Conclusion

Thus, the obtained data on the prevalence of genetic variants indicate that functional SNPs in cytokine genes are associated with asthma and various disease courses not only in adults, but also in children. However, it is evident that the results do not always agree with each other; this is due to several reasons, namely, the ethnicity of the population, the study sample size, the presence of concomitant diseases, etc. In addition, differences between children and adults can be caused by either the presence or absence, as well as the different duration of exposure of asthma patients to some environmental risk factors, including contact allergens and irritants, air pollution, smoking and occupational exposure.

An important aspect of medical practice is to achieve disease control, which is supposed to involve a personalized approach to treatment for asthma of any severity. It should be taken into account that children with severe asthma are at increased risk for adverse outcomes, including drug-related side effects, life-threatening exacerbations, and poor quality of life. As a result, the study of the distribution of allelic variants of cytokine genes in asthma among patients of different ages, representatives of different populations needs to be continued in order to find risk factors for different types of asthma. The obtained results will update with the data on the polymorphic distribution of cytokine genes in the Russian population and in asthma patients with different disease courses. This is most likely to be ultimately used for practical healthcare authorities to develop measures both in order to prevent severe uncontrolled asthma and to optimize personalized therapy.

## Conflict of interest

The authors declare no conflict of interest.
